# Mapping transmission foci to eliminate malaria in the People’s Republic of China, 2010–2015: a retrospective analysis

**DOI:** 10.1186/s12879-018-3018-8

**Published:** 2018-03-07

**Authors:** Jun Feng, Hong Tu, Li Zhang, Shaosen Zhang, Shan Jiang, Zhigui Xia, Shuisen Zhou

**Affiliations:** National Institute of Parasitic Diseases, Chinese Center for Disease Control and Prevention; Key Laboratory of Parasite and Vector Biology, Ministry of Health; WHO Collaborating Centre for Tropical Diseases; National Center for International Research on Tropical Diseases, Shanghai, 200025 People’s Republic of China

**Keywords:** Malaria elimination, Foci, China

## Abstract

**Background:**

China has initiated the National Malaria Elimination Action Plan, which aims to eliminate malaria by 2020. However, the transmission of malaria occurs sporadically or in distinct foci, which greatly hampers progress toward elimination in China and other countries. The object of this study was to foci categorization and evaluates whether the response met the requirements issued by the nation or WHO.

**Methods:**

Residual transmissions were investigated and located with fine spatial resolution mapping from parasitological confirmed malaria cases by use of routine national surveillance data. The “1–3-7” timeframes were monitored for each focus between 2012 and 2015. Each focus was identified, and the application of appropriate measures was evaluated.

**Results:**

A total of 5996 indigenous cases were recorded between 2010 and 2015; during this period, the number of cases declined by 99.1% (2010, *n* = 4262; 2015, *n* = 39). Most indigenous cases (92.5%) were reported in Anhui (*n* = 2326), Yunnan (*n* = 1373), Henan (*n* = 930), Hubei (*n* = 459), and Guizhou (*n* = 458). The temporal distribution showed that the indigenous malaria cases were clustered during the period of May to August. A total of 320 foci were carefully investigated and analyzed: 24 were active foci; 72, residual non-active foci; and 224 cleared-up foci. For the foci response evaluation, all the active foci were investigated within 7 days, while 80.2% of the residual non-active foci were responded within 7 days. In addition, reactive case detection (RACD) was carried out with 92.9% of the active foci and vector investigation carried out with 75%. For residual non-active foci, RACD was carried out with 83.2% and vector investigation with 78.2% of the foci.

**Conclusions:**

This study used nationwide data to categorize foci in China and evaluate the response of these areas during the control and elimination phases. Our approach stratifies future control responses by identifying those locations where the elimination of endemic transmission is needed, such as in the counties at the China–Myanmar border and in Tibet. In addition, this study will help local CDC staff to reassess their needs and responses against different types of foci during the elimination and post-elimination phases.

## Background

China initiated the National Malaria Elimination Action Plan (NMEAP) in 2010, which aimed to eliminate indigenous malaria in non-border areas by the end of 2015, and eliminate it nationwide by the end of 2020 [1]. All the counties in China were classified into 4 types, and each type has its own strategy and interventions [2] (Table 1).

**Table 1:** Classification of county types and strategy implemented according to NMEAP

Type	Classification	Strategy
I	Local infections are detected in 3 consecutive years, and the incident rate is equal to or greater than 1 in 10,000.	The integrated interventions of case management and vector control will be scaled up to reduce disease incidence because annual parasite incidence (API) < 1 compared to 2010.
II	Local infections are detected in 3 consecutive years, and the incident rate is lower than 1 in 10,000 in at least one of those 3 years.	Response to any possible malaria cases and active foci is the main strategy to interrupt local transmissions.
III	No local infections reported for 3 years.	Enhance monitoring and surveillance of imported cases to prevent secondary transmission.
IV	Areas without malaria epidemic.	Sensitively detect and promptly respond to imported cases.

To achieve the national goal of malaria elimination, China has made great strides in controlling indigenous malaria over the past several decades [3]. After implementing an integrated control strategy and interventions, along with socio-economic development, urbanization progress, and changes in the natural environment and malaria vectors, the incidence of indigenous malaria in China has sharply declined, and the malaria-endemic areas have dramatically shrunk [4]. Since 2010, transmissions mainly occurred in the counties along the China–Myanmar border and in Motuo County of the Tibetan Autonomous Region of China.

Malaria tends to persist in “foci” or localized areas of self-sustaining transmission, due to spatially heterogeneous influences, such as social and ecological factors that include *Anopheline* mosquito density, agricultural practices, human behavior, wealth, education, access to and utilization of health care, and urbanization [5]. In the elimination phase, cases occur sporadically or in distinct foci. In our investigation, the foci were classified into 3 types (active, residual non-active and cleared-up) according to the guidelines issued by the World Health Organization (WHO) [6]. The elimination of residual foci is a dynamic process, taking place mainly during the late stage of malaria elimination.

Monitoring the status of foci, with the precise identification of their functional status, is the cornerstone for success in interrupting malaria transmission and preventing the re-introduction of malaria where potential foci (foci with imported cases but without proof of local transmission) may be present. This step was important for China because there were many areas where transmission was sharply reduced but the vectors still exist. To interrupt malaria transmission, a two-pronged approach is required: disease management (alleviation of symptoms and prevention of biting by mosquitoes) and disease prevention through vector control.

The object of this study is to categorize foci by using country-wide data collected under operational conditions during the important period from the control phase to the elimination phase in China, and to evaluate whether the responses meet the requirements set out by national or WHO guidelines. Many factors would be challenges in the performance of the case investigation, foci investigation and response. The findings are likely to reflect the real situation and would help local CDC staff to reassess their needs and responses against different types of foci during the elimination and post-elimination phases.

## Methods

### Study design

A retrospective study was conducted to explore malaria-endemic characteristics from January 1, 2010 to December 31, 2015 at the Chinese Center for Disease Control and Prevention (CDC) [7–12]. All individual cases from the Infectious Diseases Information Reporting Management System (IDIRMS, http://chinacdc.cn) were carefully reviewed and analyzed [13]. The IDIRMS, which was set up in 2004 after the SARS outbreak, is a standardized platform that provides health care systems nationwide the ability to detect, analyze, prevent, and respond to any communicable disease in the country. The data for the study were selected by use of the reporting data and reporting area, but the data from Hong Kong, Macao, and Taiwan were excluded from these statistics. The IDIRMS parameters consisted of the geographical distribution, species composition, gender, and age distribution of the cases. Both clinically diagnosed cases and laboratory-confirmed cases were included in this analysis (Table 2).

**Table 2:** Definitions used in this study

Type of malaria	Description
Clinically diagnosed case	An individual with malaria-related symptoms (fever [axillary temperature ≥ 37.5 °C], chills, severe malaise, headache, or vomiting) at the time of examination.
Laboratory-diagnosed case	A clinical case confirmed by microscopy, polymerase chain reaction, or rapid diagnostic tests in the laboratory.
Indigenous case	A case contracted locally with no evidence of importation and no direct link to transmission from an imported case. In this study, an indigenous case refers to malaria acquired by mosquito transmission in China.
Imported case	A malaria case or infection in which the infection was acquired outside the area in which it was diagnosed. Here, it refers to the patient who acquired the illness from a known malaria-prevalent region outside China.
Induced case	A case in which the origin of the illness can be traced to a blood transfusion or other form of parenteral inoculation of the parasite but not to transmission by a natural mosquito-borne inoculation.
Introduced case	A case contracted locally, with strong epidemiological evidence linking it directly to a known imported case (first-generation local transmission).
Recrudescent case	Recurrence of asexual parasitemia of the same genotype(s) that caused the original illness, due to incomplete clearance of asexual parasites after antimalarial treatment.
Death from malaria	Patient with signs and symptoms of complicated malaria, with confirmed diagnosis of *P. falciparum* (or *P. vivax*) or associated infection.
Focus	A defined and circumscribed area situated in a currently or formerly malarious area that contains the epidemiological and ecological factors necessary for malaria transmission.
Reactive case detection	A process that involves an active response after the identification of a local or imported case in a receptive area where the transmission intensity is low or assumed to be interrupted.

Another reporting system, the Parasitic Diseases Information Reporting Management System (PDIRMS, http://chinacdc.cn), was set up in 2012 to report on 3 diseases: malaria, schistosomiasis, and echinococcosis. Malaria was monitored and reported on within the time frame indicators using the “1–3-7” strategy. The “1–3-7” strategy, which refers to the reporting of malaria cases within 1 day, case confirmation and investigation within 3 days, and foci investigation and response to prevent further transmission within 7 days, was launched in 2012 to guide and monitor the elimination process nationwide [14]. Each case was investigated by staff at the county CDC to classify it as indigenous or imported; the definitions appear in Table 2 [15]. The PDIRMS contains the date of diagnosis, date of reporting, date of the case investigation, case classification (indigenous, imported, or other [induced, introduced, or recrudesce]), focus investigation, and foci response, such as reactive case detection (RACD) and indoor residual spraying (IRS). Both the website for the IDIRMS and the PDIRMS are private. Malaria department in NIPD was responsible for national malaria data for these two systems and have permission to access them.

### Data extraction for foci identification and response evaluation

In this study, a natural village is considered the smallest unit of focus. “Active focus” refers to a focus with ongoing transmission; “residual non-active focus” refers to a transmission that was interrupted recently (1–3 years ago). A “cleared-up focus” is defined as a focus with no local transmission for more than 3 years.

For foci identification, the data of the indigenous cases from 2012 to 2015 in the IDIRMS were carefully reviewed and analyzed. Because it was not possible to distinguish the precise foci location for domestically mobile cases within the country (these cases are also defined as indigenous cases), only the foci with exact information were selected for categorization. If 2 or more cases were reported at 1 focus, they were considered and recorded as only one focus because the aim of this study is to classify foci and evaluate the response strategy. For the foci response evaluation, only the indigenous cases from the IDIRMS that matched the data in the PDIRMS were selected and analyzed. The population data for every county in China from 2010 to 2015 were obtained from the National Bureau of Statistics of China (http://data.stats.gov.cn/). All malaria cases reported were geo-coded and matched to the county-level layers of polygon and point using the software ArcGIS 10.1(Environmental Systems Research Institute, Inc., Redlands, CA).

## Results

### Indigenous malaria in China, 2010–2015

From 2010 to 2015, 5996 indigenous cases were recorded by the IDIRMS. During this period, the reported indigenous cases sharply declined by 99.1% (2010, *n* = 4262; 2015, *n* = 40). Since data of indigenous malaria cases were not available for 2010–2011, the epidemiological data for *Plasmodium* species from 2012 to 2015 were collected. These data were analyzed, and the results indicated that most of the indigenous cases (63.3%) were attributed to *P. vivax*. Like *P. vivax,* the number of local *P. falciparum* cases declined from 97 in 2010 to 1 in 2015 (this strain only occurred in Yunnan Province).

Transmission largely decreased during the study period; indigenous cases occurred in 303 counties in 18 provinces in 2010, while in 2015, transmissions occurred in only 9 counties in 4 provinces (Table 3).

**Table 3:** Indigenous cases in China, 2010–2015

Year	Total cases	Indigenous (%)	Local *P. v* (%)	Local *P. f* (%)	No. of counties with transmissions	No. of Type I^a^ counties with transmissions	No. of Type II^b^ counties with transmissions
2010	7855	4262 (54.3)	NA^c^	97 (2.3)	303	71	203
2011	4498	1308 (29.1)	NA^c^	32 (2.4)	155	60	92
2012	2718	244 (9.0)	228 (93.4)	16 (6.6)	41	30	9
2013	4128	86 (2.1)	77 (89.5)	9 (10.5)	12	9	3
2014	3078	56 (1.8)	45 (89.3)	6 (10.7)	10	9	1
2015	3116	39 (1.2)	38 (97.5)	1 (2.5)	9	8	1
Total	25,393	5995 (23.5)	393	32	530	187	309

^a^Type I counties refer to areas with local infections that are detected in 3 consecutive years with an incidence rate equal to or greater than 1 in 10,000

^b^Type II counties refer to areas with local infections that are detected in 3 consecutive years with an incidence rate lower than 1 in 10,000 in at least 1 of those 3 years

^c^NA indicates that data were not available in the annual reporting system

**Fig. 1:**
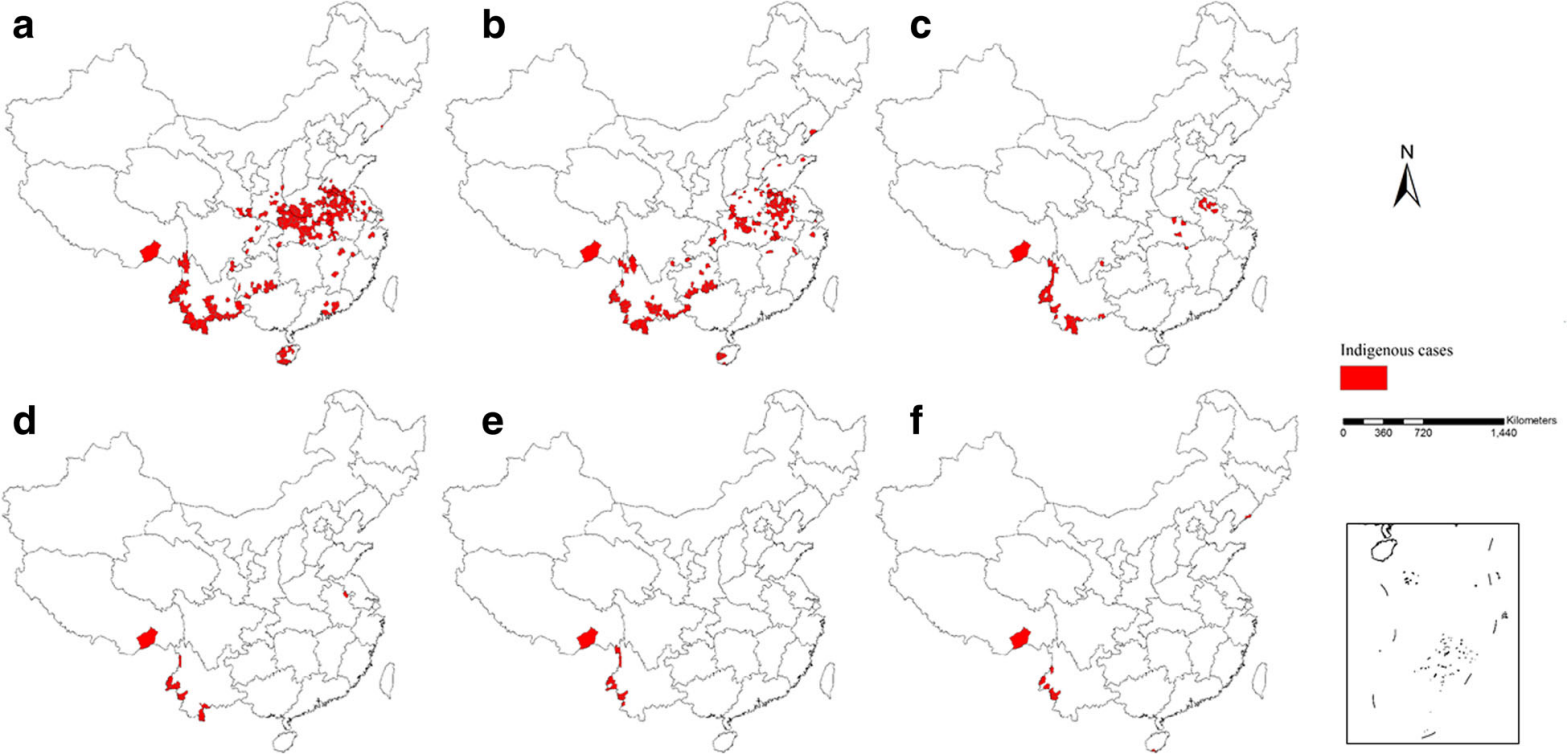
Indigenous malaria in China, 2010–2015. **a** 2010, **b** 2011, **c** 2012, **d** 2013, **e** 2014, and **f** 2015. The red zones represent the areas where indigenous cases occur (county level)

In this study, indigenous cases occurred in 93.6% of the Type I and Type II counties. From 2010 to 2015, transmissions in Type I and Type II counties significantly decreased, particularly for Type II counties, while in 2015 only 1 Type II county in Dandong city, Liaoning Province, reported indigenous cases (*n* = 2; Fig. 1).

**Fig. 2:**
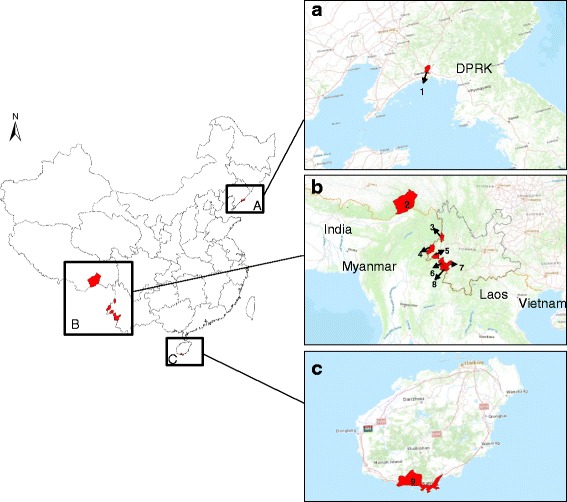
The foci at county-level and its neighboring countries reported in 2015. **a** 1 represents Donggang County in Liaoning province neighboring with Democratic People’s Republic of Korea (DRPK), **b** 2 represents Motuo County in Tibet, 3, 4, 5, 6, 7, 8 represent counties of Lushui, Yingjiang, Mangshi, Zhengkang, Gengma, Cangyuan in Yunnan province, respectively, where neighboring with Myanmar, **c** 9 represents Sanya County in Hainan province

Most indigenous cases (92.5%) were reported in Anhui (*n* = 2326, 38.8%), Yunnan (*n* = 1373, 22.9%), Henan (*n* = 930, 15.5%), Hubei (*n* = 459, 7.7%), and Guizhou (*n* = 458, 7.6%). However, in Liaoning and Hainan, where local transmission was blocked for 4 years, officials reported 2 local *P. vivax* and 6 local *P. malariae* cases in 2015 (Fig. 2).

### Temporal distribution of indigenous malaria

**Fig. 3:**
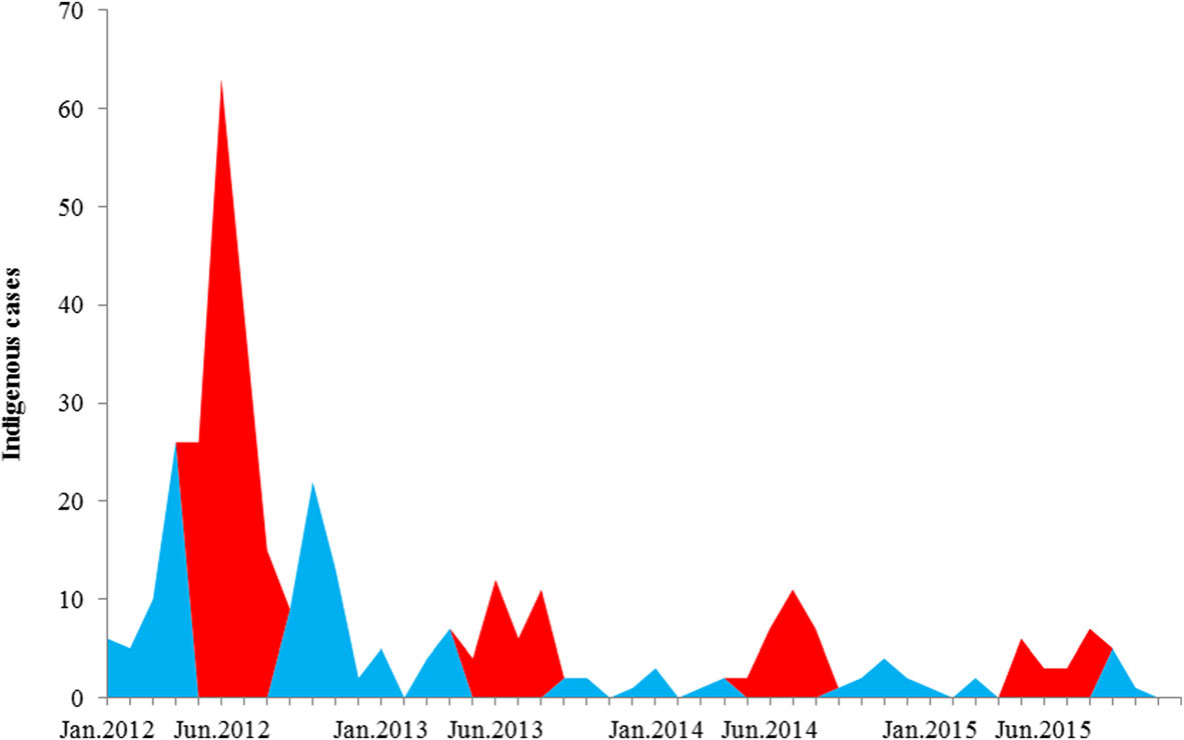
Temporal distribution of indigenous malaria, 2012–2015. Red zones represent the period from May to August, and blue zones represent the period from September to April (the next year)

**Fig. 4:**
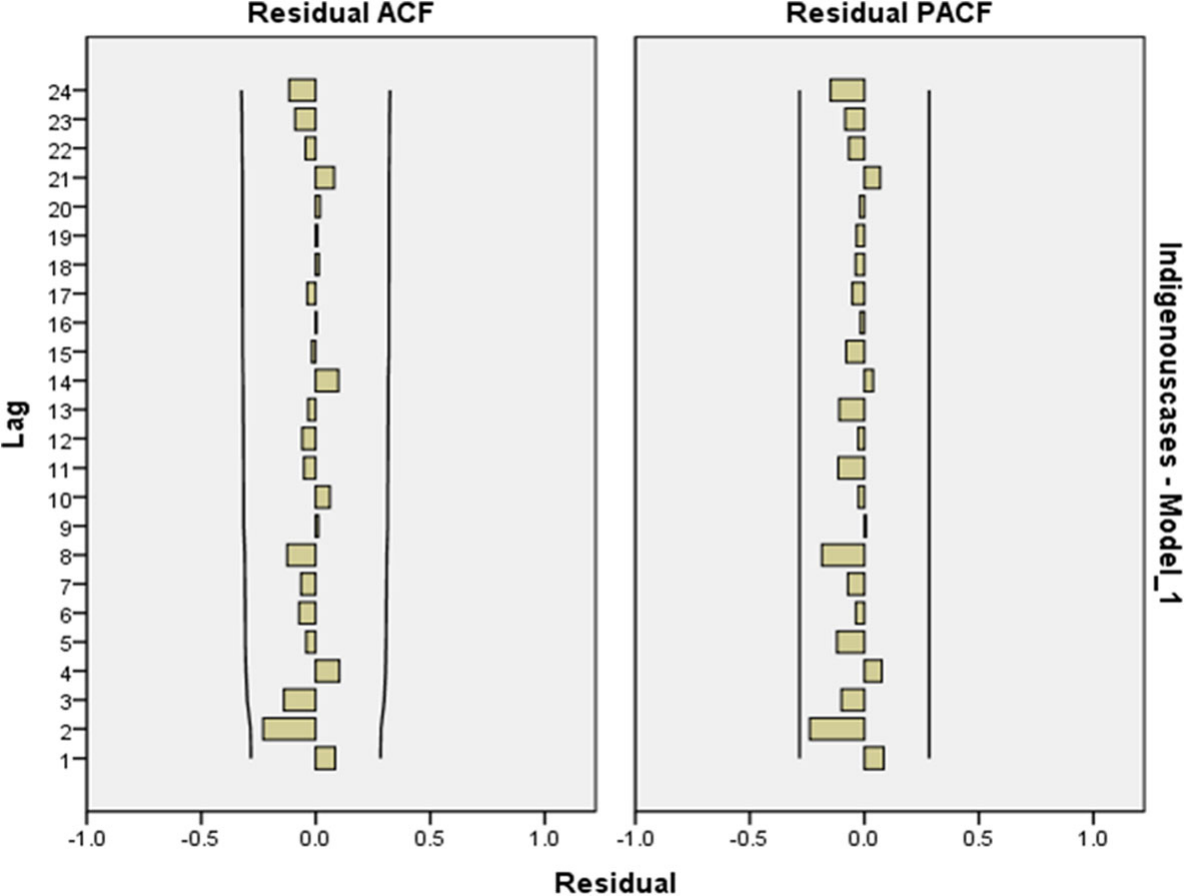
Residual ACF and residual PACF of indigenous malaria, 2012–2015

The temporal distribution showed that indigenous malaria clustered between May and August. The highest number of transmissions was reported in June 2012, with 63 indigenous cases. The indigenous cases have sharply decreased since 2013: for example, only 12 indigenous cases were reported between May and August 2015, a 92.7% reduction compared with the same months in 2012 (Fig. 3). The residual auto correlation function (ACF) and residual partial correlation function (PACF) for the indigenous model also proved that the summer season (May–August) was the time frame with the most reported cases (Fig. 4).

### Foci identification and classification

**Fig. 5:**
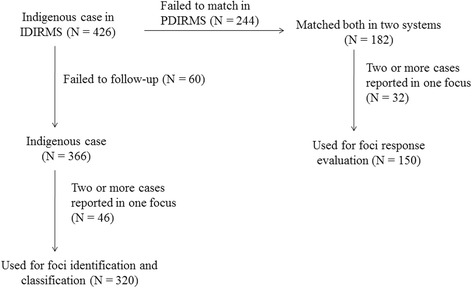
Data flow for foci identification and response evaluation

A total of 426 indigenous cases that were reported between 2012 and 2015 were carefully investigated and analyzed for foci identification and classification. Despite some unclear foci investigations (*n* = 60) and 2 or more cases reported in 1 focus (*n* = 46), 320 foci were finally obtained and used for foci identification (Fig. 5).

The 320 foci were investigated and classified as 24 active foci, 72 residual non-active foci, and 224 cleared-up foci. The 24 active foci were distributed in 6 counties of Yunnan, 1 county of Hainan, 1 county of Liaoning, and 2 counties of Tibet (Table 4). The 72 residual non-active foci were distributed across 15 counties of Yunnan, 2 counties of Tibet, and 2 counties of Anhui.

**Table 4:** Active foci in China (the foci that were investigated in each natural village^a^)

Foci No.^b^	Province	County	Township	Village	Case
1	Yunnan	Yingjiang	Nabang	Jiedao	2
2				Nabang	1
3				Lishu	1
4				Daonong	1
5				Qiaotou	1
6			Kachang	Caobei	1
7		Gengma	Mengding	Hanhong	1
8				Qiushan	1
9		Mangshi	Xuangan	Qincaitang	1
10			Santaishan	Yunqian	1
11		Zhengkang	Mengdui	Yakou	1
12		Lushui	Pianma	Pianma	1
13		Canyuan	Banlao	Banlao	1
14	Tibet	Motuo	Beibeng	Gelin	1
15				Jiangxin	1
16				Beibeng	1
17			Motuo	Yadong	1
18				Bodong	1
19			Dexing	Dexing	1
20		Linzhi	Bayi	Yingbin	1
21	Hainan	Sanya	Fenghuang	Lixin	4
22				Baolong	2
23				Nandao	1
24	Liaoning	Donggang	Jiangzi	Zhangdao	2

^a^A natural village has approximately 50 households with a population of 200 to 250 people and is the lowest administrative level in China. In China, a natural village with a reported malaria case is considered a focus

^b^If 2 or more cases were reported at 1 focus, only 1 focus was recorded. In this study, more than 1 case was reported in Jiedao, Lixin, Baolong, and Zhangdao; therefore, those 4 sites were considered as 4 foci

### Foci response evaluation

For the foci response evaluation, 43.9% (*n* = 182) of the 426 indigenous cases selected from the IDIRMS were matched in the PDIRMS (Fig. 5). Further analysis found 32 of them were classified as 2 or more cases reported in 1 focus; therefore, 150 foci were evaluated for foci response.

In 2015, 28 indigenous cases were reported and foci response was carried out. All the active foci were investigated within 7 days; 92.9% (*n* = 26) of foci carried out RACD, and 75% (*n* = 21) carried out vector investigations (Table 5). Two positive patients were screened by RACD and found to have *P. vivax* (Yingjiang County) and *P. malariae* (Sanya County). A total of 101 residual non-active foci were evaluated, and most of them (*n* = 81, 80.2%) responded within 7 days (Table 5). In addition, 83.2% (*n* = 84) of the foci carried out RACD, and 78.2% (*n* = 79) carried out vector investigations. Four people were diagnosed as malaria-positive by RACD, and 83.2% (*n* = 84) of the foci carried out IRS. For the cleared-up foci, 21 foci were matched in both systems: only 1 focus investigated and responded within 7 days, while 16 carried out RACD and 19 conducted vector investigations (Table 5).

**Table 5:** Evaluation of foci response for the 3 types of foci in China

Response	Foci classification		
	Active^a^	Residual non-active^b^	Cleared-up
Total cases	28	101	21
Population at risk^c^	6452	46,809	10,006
No. of foci responding within 7 days (%)	28 (100)	81 (80.2)	1 (4.8)
No. of foci that carried out RACD (%)	26 (92.9)	84 (83.2)	16 (76.2)
Screened population	1447	2985	712
No. of malaria-positive patients	2	4	0
No. of foci that carried out entomological investigations (%)	21 (75)	79 (78.2)	19 (90.5)
No. of foci that carried out IRS	26 (92.9)	84 (83.2)	18 (85.7)

^a^All the indigenous cases reported were considered active foci. In 2015, 28 indigenous cases were reported, and foci response was carried out for all of them

^b^A total of 101 cases reported from 2013 to 2014 were categorized as residual non-active foci

^c^The population at risk represents the recorded data for each type of foci

## Discussion

During the elimination phase, population-level measures become inefficient and inadequate, so as countries approach the goal of eliminating malaria, individual-based estimates of transmission must identify foci where resources should be targeted because transmission remains high [16]. Aggregate ratios of indigenous-to-imported cases in time (or in space) alone, could obscure localized transmission if, for example, most cases failed to transmit but some pockets of transmission remain.

The border areas are a great challenge for malaria elimination in China because they harbor a mixed endemic region with both *P. falciparum* and *P. vivax* malaria transmission [17]. The natural environment in these areas is complex and a variety of malaria vectors, such as *Anopheles dirus* and *An. minimus*, usually co-exist in a single setting and have a high vector capacity for transmitting malaria [18]. In addition, there is a large mobile cross-border population since there is no natural barrier in this region, which makes management of imported malaria a significant challenge [19–21]. Poor transportation also makes it difficult to conduct epidemiological studies and blood smear verification within 3 days [22]. In this study, the healthcare workers’ response in these foci was inefficient, although all the cases at the foci were treated within 7 days. First, not all the active foci carried out RACD and IRS, which are required by the national strategy. Second, only three-quarters of the active foci conducted a vector investigation; without favorable entomological information, 2 potential risks remain. One is that the imported *P. vivax* can be re-introduced under conducive ecological conditions (transmission season) if the area harbors an appropriate vector, although there is little evidence of re-introduction of imported malaria in China [23]. Another is the increasing importation of *P. falciparum* to China due to a lack of knowledge about the efficient vector capacity to sustain imported *P. falciparum* for the re-establishment of transmission. A foci investigation consists of an assessment of potential *Anopheles* breeding sites, the collection of adult mosquitoes to identify the species responsible for transmission, and the assessment of the vector’s susceptibility to insecticides; these steps are absent in routine surveillance work, but they are required by national guidelines. In addition, 2 malaria-positive patients who were screened by RACD revealed an insensitivity for passive case detection, this insensitivity is a particularly difficult issue for some regions where transmission was absent for several years, such as Hainan, where there was no transmission in 2011. However, in 2015, 7 indigenous cases were reported in this province [24]. This resurgence shows the significance of surveillance systems during the elimination stage, and especially in the post-elimination stage, to monitor for the potential re-introduction of malaria into these areas.

The active foci response required a strategy that combined RACD with vector control to clear the foci [25]. Given proof that asymptomatic people are present at the China–Myanmar border [26], a more sensitive technology is required to screen local residents and frequently mobile populations, including temporary workers and illegal immigrants, who may not routinely use established health services. In China, RACD was performed in the households where a case was identified and neighboring households within a 300-m radius if the focus was considered large (an entire village) [27]. The RDT was adopted in the field and all blood samples were taken and sent to the provincial CDC or national CDC for PCR verification. Currently, the CDC staff at the China–Myanmar border use a high-throughput, low-cost, and highly sensitive screening method based on 18S ribosomal RNA to detect asymptomatic sub-patient infections. In 96-well plates, the samples are quantified by the amount of ligated probes that bind continuously to the 18S rRNA of the genus *Plasmodium*; this method may offer an alternative for sensitive, large-scale molecular screening that can be used for RACD [28].

All the measures implemented in these foci, e.g., RACD, IRS, targeted mass drug administration (tMDA), and health education, should follow the national guidelines because they significantly control the foci in the next year. Malaria elimination programs follow-up malaria cases reported by health facilities in order to carry out case investigations that will determine the origin of the infection, whether it has been imported or is due to local malaria transmission. All the active foci should carry out RACD and IRS to reduce the reservoir of asymptomatic and low-density infections, and prevent mosquitoes from biting to block transmission. Targeted interventions such as tMDA may be considered for patients, tMDA was performed on a smaller scale, used for close contacts, households, and villages of index cases.

The residual non-active foci in China cover 19 counties in 3 provinces. Not all the foci investigated and/or responded within 7 days because of poor transportation in Yunnan and Tibet, which caused delays for the CDC staff. To solve this problem, the government has improved road infrastructure; thus, the CDC staff could arrive at the foci in a timely manner. For remote areas, provincial CDC staff designated a central location at which they carry out to carry out case diagnosis, investigation, and treatment. For example, Yunnan Institute of Parasitic Diseases has set up a work station in Nabang village (one village of Yingjiang County, neighboring Myanmar) to conduct case diagnosis by use of PCR technology during the transmission season.

Despite a lack of indigenous cases occurring at these foci, there is still the possibility of relapse for *P. vivax* and *P. ovale*. A relapse would require that the local CDC staff emphasize the quality of treatment when visiting each house at the time of radical treatment. An attempt should be made to contact people who were absent during the healthcare visit. Although it is hard to carry out in the primary health care sectors, especially with minority populations who were present at the China–Myanmar border and in Tibet, the use of glucose-6-phosphate dehydrogenase deficiency assays should also be considered. In China, people who relapsed with *P. vivax* and *P. ovale* should undergo directly observed therapy, taking primaquine for radical treatment [29]. After 1 or 2 years without evidence of transmission, the 72 residual non-active foci may be re-categorized as cleared-up foci.

In addition, we have also summarized the similarities and differences between the frameworks of the WHO and China. While both frameworks could guide classification of foci, WHO’s definition and identification of foci mainly classifies foci by tracking them over the last 3 years, and China investigates the response of foci to any current cases, including imported cases. A natural village with any reported cases was assumed to be a focus and was classified based on the *Plasmodium* species, season, and vectors. Once an imported case was reported, it was checked by microscopy or PCR in a reference lab to determine the *Plasmodium* species, and then the case was investigated to determine whether it occurred during transmission season. For example, if imported *P. vivax* was reported during transmission season, the CDC staff would carry out a response appropriate for the type of focus, including RACD and IRS, combined with tMDA if it was an active focus. Unlike the WHO classification, the purpose of foci classification in China is to allow the CDC staff to determine the appropriate response so that each patient can be identified early and obtain appropriate treatment.

### Limitations

Firstly, not all indigenous cases were well recorded for the exact epidemiological information, especially for domestically mobile (within the country) cases; in this study we missed 60 indigenous cases information. Secondly, the 2 web-based reporting systems were set up in different years, which led to a mismatch in the cases posted in these systems. This issue is also a problem with the PDIRMS, which was established in 2012. More time should be invested in integrating the IDIRMS data with the PDIRMS data and in training local CDC staff. Thirdly, we did not know the exact reasons for the delay in foci investigation and response though these are relatively minimal, they still need specific correction. Fourthly, because the ArcGis software used for foci mapping at village level is not presently available, and because the presentation of all foci in one map would give a low-resolution picture due to the number of foci (more than 200 foci in 2012 and dozens between 2013 and 2015), in this report we provided the indigenous cases at county level from 2010 to 2015.

## Conclusion

This study used nationwide data to categorize foci in China and evaluate the response of these areas during the control and elimination phases. Our approach stratifies future control responses by identifying those locations where the elimination of endemic transmission is needed, such as in the counties at the China–Myanmar border and in Tibet. In addition, this study will help local CDC staff to reassess their needs and responses against different types of foci during the elimination and post-elimination phases.

## Abbreviations

ACF: Residual auto correlation function
CDC: Center for Disease Control and Prevention
IDIRMS: Infectious Diseases Information Reporting Management System
IRS: Indoor residual spraying
LLTN: Long-lasting insecticidal nets
NMEAP: National Malaria Elimination Action Plan
PACF: Residual partial correlation function
PCR: Polymerase chain reaction
PDIRMS: Parasitic Diseases Information Reporting Management System
RACD: Reactive case detection
tMDA: targeted mass drug administration
WHO: World Health Organization
